# Tin and Gold Fillings

**Published:** 1888-09

**Authors:** W. D. Miller

**Affiliations:** Berlin


### Tin and Gold Fillings.

DR. W. D. MILLER, BERLIN.

In the April number of the DENTAL REGISTER, pages 191-2 may be found the following statements:

"*Dr. Conrad*—Said that a short time since Dr. Eames had a patient of Dr. Herbst's. There were about fifty of these combination fillings in the mouth. In ten or twelve the tin had disappeared entirely, in two or three partially, and the other fillings were in such a condition that they all had to come out. The failure was attributed to the kind of tin used in the operations.

*Dr. Watt*—Said that in some cases the action brought about by the combination caused uneasy sensations or pain in the tooth and sometimes so severe as to cause sleeplessness He stated that it was not thoroughly understood just what the trouble was, but he was of the opinion that it was thermal electricity.

*Dr. McKellops*—Had seen much of the combination filling, but there was nothing that gave him more satisfaction than gold. He thought non-cohesive gold was just as adaptable as tin or tin and gold. This could be proven by experiment. Take tin and adapt it to the wall of a cavity, and then adapt pure gold to the same wall, and you will perceive that the hair lines on both are precisely alike."

To these statements permit me to answer first: no kind of tin foil I ever saw, not even that which comes around soft cheese is bad enough to account for the failures mentioned by Dr. Conrad. *Rotation did it.* The first layers of tin-and-gold may be pressed against the walls of the cavity by a rotating burnisher or by a hand burnisher, but any one who attempts to complete a tin-and-gold filling by rotation alone, must I fear, expect the results recorded by Dr. Conrad.

I do not at all agree with, in fact I most emphatically disagree with Dr. Watt's statement. Having used tin-and-gold in over ten thousand cases, and seen nearly as many more where it had been used by Drs. Abbott, Jenkins, Sachs, and others, I have in not a single case observed any uneasy sensations characteristic of this combination.

Dr. McKellop's experiment is not a test of adaptability, although curiously enough it has been looked upon as such by dentists for many years.

According to this test extra-cohesive gold foil is more adaptable than either non-cohesive gold foil or than tin foil, while rubber or coffer-dam, which we know is an excellent material for obtaining a water-tight joint, is absolutely non-adaptable. We can obtain a beautiful impression of a coin with extra-cohesive gold foil, a less perfect impression with non-cohesive or tin foil, and no impression at all with rubber. This experiment indicates simply the property of retaining an impression once given. Adaptability must be tested in altogether a different manner. Having used both non-cohesive gold and tin-and-gold very extensively, I do not hesitate to attribute a higher degree of adaptability to the latter material.

Candahol as a Local Anæsthetic.—Dr. Phouchkine recommends candahol, a hydrocarbon distilled from the American naphtha, as a local anæsthetic. It is a transparent liquid insoluble in water or alcohol, and produces complete anæsthesia in sixty seconds. It is available for the removal of ingrowing nails, in whitlow, the enucleation of cysts. Another advantage of candahol is its moderate price.

Skin Grafting.—Skin grafting is now quite common for covering the expansive wastes of old leg ulcers, and other parts of the body denuded of the natural covering. But is is not now transplanted by the square inch; the smaller the graft the better. Here is one way of doing it: Insert a delicate needle under the epidermis of some healthy spot, and after passing a small pair of curved scissors or a sharp scalpel under the needle, snip off a minute portion of the epidermis. Carry this on the needle to the denuded part, and press it lightly down, taking care that the graft rests on its fresh surface.

A Japan Giantess, according to native journals, though only twelve years and five months of age, stands eight feet high, and weighs 270 pounds; her hands are nine inches.—*Scientific American.*

